# From Opioid Pain Management to Opioid Crisis in the USA: How Can Public-Private Partnerships Help?

**DOI:** 10.3389/fmed.2019.00106

**Published:** 2019-05-14

**Authors:** Larissa Bolliger, Hilde Stevens

**Affiliations:** ^1^Department of Public Health and Primary Care, Ghent University, Ghent, Belgium; ^2^Institute for Interdisciplinary Innovation in healthcare (I3h), Université libre de Bruxelles, Brussels, Belgium

**Keywords:** opioid crisis, pain management, public-private partnerships, USA, a perspective

## Abstract

The current opioid crisis in the USA arose from (at first) successful opioid pain management in three waves, starting in the'90s. Today, USA patients consume opioid drugs on a massive scale. Considering their potential for tolerance, as well as their potential for lethality in relatively small overdose, the overuse of opioids form an urgent threat to public health in the USA. Since the opioid crisis is a complex phenomenon, several stakeholders are needed to tackle the problem. Both public and private stakeholders should collaborate, e.g., in Public-Private Partnerships. Those collaborations should focus on different aspects related to the opioid crisis such as medical and societal (e.g., pain management process, including addressing opioid use disorders), as well as economical and regulatory issues (e.g., incentivizing the search for alternative non-addictive pain medication and banning aggressive marketing tactics used by the pharmaceutical industry). Additionally, collaborations should cover interdisciplinary education and training of various healthcare actors involved. In conclusion, interdisciplinary collaboration on the various opioid abuse-related aspects is urgently needed to tackle the opioid crisis in the USA.

The opioid crisis is a byproduct, and more particularly, an adverse event of medical care (1).

The “opioid drug” class contains four categories according to the Centers for Disease Control and Prevention (CDC): (1) natural opioid analgesics, such as morphine and codeine as well as the semi-synthetic opioid analgesics such as oxymorphone, hydrocodone, oxycodone, and hydromorphone, (2) the synthetic opioid methadone, (3) any other synthetic opioid, such as fentanyl and tramadol, and (4) the illegally-made opioid heroin, synthesized from morphine (2). Legally produced opioids are safe, highly effective analgesics and indispensable in modern pain management, when used for a rightful purpose, during supervised therapy and handled by a competent physician (3).

In 1986, the World Health Organization (WHO) proposed a three-step pain ladder for the treatment of pain (4). The first step comprises non-opioid analgesics, such as paracetamol, aspirin, and non-steroidal anti-inflammatory drugs (NSAIDs) for managing mild pain. In the second step come weak-opioids like codeine or tramadol for moderate pain. The third step includes strong opioids such as morphine, fentanyl, and oxycodone (5). Opioids should only be prescribed when non-opioid analgesics and adjuvant therapies were unsuccessful. Additionally, opioids should be dosed as low as possible, achieving pain relief with a minimal level of side effects (3).

Dependence is a serious side effect associated with opioid use, which can lead to compulsive use despite negative consequences, a principal characteristic of substance abuse disorders as defined by the Fifth Edition of the Disease and Statistical Manual of Mental Disorders (DSM-5). Opioid use has led to a public health crisis which is characterized by an exponential growth in people suffering from opioid use disorders in several countries, notably the USA. This opioid crisis has been described as the latest self-inflicted threat to public health in the USA where drug overdoses, predominantly caused by opioids, are the leading cause of death for people under the age of 50 (1).

The USA opioid crisis rose from a perfect storm of events with three major waves (Graph 1) (6). Since the 1990s, a first wave resulted in increased deaths related to natural and semi-synthetic prescription opioids (7). Since 2010, a second wave was observed with a rapid increase in deaths caused by heroin overdoses. Since 2013, a third wave began with overdose deaths involving synthetic opioids, especially fentanyl (8). Together, this has led to an exponential increase in deaths related to opioid overdose and it has been widely debated what caused those waves.

The opioid crisis has been proposed to initially stem from efforts to address the problem of under-treatment of pain, which motivated practice and policy shifts (9). In the early 1990s, the number of opioid prescriptions increased consequently, not only for the treatment of acute pain (10) but for chronic pain as well (11). Given that up to eleven percent of chronic pain patients using opioids were found to meet criteria for substance abuse disorders (12), these practice and policy shifts may readily explain the steady growth in prescription opioid abuse. This was exacerbated by other factors. Pharmaceutical companies interested in expanding their markets misused the practice and policy shifts with aggressive marketing strategies (13, 14). In 1996, an extended-release oxycodone formulation was introduced to the market that was proposed by the manufacturer to be effective for 12 h while being less addictive (15). At the same time, when patients still experienced pain, they were advised to take higher doses, rather than taking the extended-release oxycodone more frequently (16). This further nurtured so called “pill mills,” physicians who prescribe opioids regardless of medical need (17). Moreover, not all prescription opioids are taken by pain patients. Particularly acute pain patients take on average only one third of the prescription opioids (18). Each of those leftover pills can be sold with substantial financial profit, leading to an increased availability of prescription opioids for non-medical use. Due to the disappearance of manufacturing jobs, rising inequality, the economic crisis in 2008 and long-term unemployment, the temptation to sell the drugs for cash or to take them for emotional relief instead of physical pain, increased. Moreover, some of the prescription opioid users discovered that the time-release mechanism of slow-release oxycodone could be defeated by crushing the pills and injecting or snorting the now effectively short-acting and highly addictive opioid. Discussions about how to misuse the prescription opioids and their effects became public and spread quickly, especially through the growing use of the internet (16). In 2010, the government started to crack down on “pill mills” (17). Additionally, an abuse deterrent formula for slow-release oxycodone was announced as an attempt to meaningfully deter abuse by making it more difficult to crush the pills (19). As a result of those actions, some of the people addicted to prescription opioids changed to using heroin, since it is easier to use and cheaper (20). Indeed, as much as eighty percent of heroin users started with taking prescription opioids (21). Compared to heroin, synthetic opioids are relatively easy to produce and traffic in substantial quantities given their very high potency. It is therefore not surprising that from 2014 onwards, the number of fentanyl-related deaths increased by 72% (22).

Pharmaceutical companies provide physicians with free drug samples, including prescription opioids, for promotion purposes. This is a common practice in the USA. While in the European Union (EU), opioids are subject to legal measures since 1992, so far, no initiative to ban the provision of free samples has been introduced in the USA. Even though the difference in drug marketing can be linked to different health care systems between the EU and the USA, banning free drug samples should be urgently considered by policymakers (13, 14). By now, opioids belong to the most widely-prescribed drug classes in the USA. The country faces an extreme situation since it constitutes only five percent of the world's population, but it consumes 56% of the world's opioid drugs (23).

## Multi-stakeholder Collaborations Needed to Tackle the Opioid Crisis

Opioid analgesic abuse is a complex and multifactorial problem. No single stakeholder can solve this crisis independently (24). In 2005, the Food and Drug Administration (FDA) transformed the drug risks and benefits assessment and issued a series of guidance and the so called “risk minimization action plans” (RiskMAPs) including risk minimization tools offered to industry in order to achieve “specific health outcomes related to known safety risks” (25). In 2016, the FDA issued its Opioids Action Plan (26), aiming to reduce opioid prescription behavior through education programs. The corporate social responsibility of the pharmaceutical industry in the USA lacks. In its Opioid Action Plan, the FDA also aims to reexamine the underlying risk-benefit paradigm for opioids (14, 27). The pharmaceutical industry should contribute with its expertise in product abuse risk assessment, product communication and education, and cooperate with federal and state authorities, as well as with law enforcement authorities (24). They should implement product abuse and diversion procedures in their corporate mission statement, comparable as what the European Federation of Pharmaceutical Industries and Associations (EFPIA) have introduced in the healthcare professional code (28).

Already in 2003, collaborative initiatives had been set up to unite diverse investigator groups to cooperate in the field of pain research, such as the Brain Research through Advancing Innovative Neurotechnologies (BRAIN). Examples of pain management partnerships include the Multidisciplinary Approach to the Study of Chronic Pelvic Pain (MAPP) aiming to define an imaging-based signature for pelvic pain, or the Collaborative Health Outcomes Information Registry (CHOIR) wherein an open-source learning healthcare platform was used as a basis to develop a deep signature of individual patients across several dimensions of social, psychosocial, and physical functioning. This registry generated new insights about what leads to pain persistence and what drives pain in general. However, the setup of those partnerships was not broadly implemented (22) and certainly did not end the trend of the significant increase in non-medical use of prescription opioid analgesics in the USA.

In order to boost collaboration, President Obama dedicated in 2016 more than 1 billion US Dollars to set up evidence-based prevention programs to support monitoring of prescription drugs, take-back events related to prescription drugs, and to facilitate the access to the overdose reversal drug naloxone (29).

Tackling the prescription opioid abuse requires a joint effort of multiple stakeholders involved in the pain management process. Public-private partnership (PPP) models vary depending on the type of participants, the funding, the mission, and objectives (30).

PPPs such as the International Rare Diseases Research Consortium (IRDiRC) (31) could serve as a role model in the fight against opioid addiction. The FDA could benefit from close collaboration with industry to review the system barriers and organizational issues in the regulatory system and work toward official action to end the practice of aggressive marketing strategies. The Orphan Products Grants Program of IRDiRC integrates industry, regulatory agencies and patient advocacy organizations to collaboratively develop recommendations for improved R&D and guidelines, regulation and patient involvement. The FDA jointly works with industry to define incentives including expedited reviews of new drug applications for alternative products, having tamper-resistant properties and support in the development of risk management plans (24). Furthermore, the reimbursement policies could be a subject of the regulatory science-based consortia, aiming to prevent addicted patients to turn to cheaper, but illegal alternatives which are more lethal.

Collaborations should focus on various aspects related to the pain management process, including addressing the industry's lack of interest to invest in developing non-addictive pain medication and addressing opioid use disorders. Since deterring data concerning the development of successful and failed pain medication is one major stumbling block (32), PPPs can be set up that focus on more precompetitive targets, such as the development of data management tools, registries, and shared databases. These data could facilitate the understanding of heterogeneous patient groups and their characteristics when integrated in clinical trials. Also developing biomarkers to stratify patients for trials and demonstrating target engagement, applying new technologies to facilitate pain medication discovery, creating a research trial network, establishing objective pain sensitivity measures, and reengineering preclinical platforms to improve predictive efficacy are topics that can be the focus of early-phase research PPPs. Further, developing subgroups of large cohort studies and repurposing already existing compound, doing molecular profiling to begin validating targets and working on new chemical entities, developing new bioinformatics tools for target discovery purposes and reverse and forward translation, cellular and mechanistic studies are situated in the precompetitive sphere. Another aim of PPPs could be to screen research using preclinical addiction models with focus on reproducibility and reliability of published data. Moreover, PPPs might do research on pharmacogenetics of addiction and focus on working on programs to deliver extended-release formulations of buprenorphine (opioid to treat opioid addiction) in hospital emergency units to prevent opioid overdose, drug-seeking, and drug-taking behavior (32).

Depending on the objectives of the partnership, public and private constituencies affected by this societal crisis need to collaborate, such as healthcare practitioners, patients, professional organizations, researchers, educators, pharmaceutical manufacturers, insurers, public health agencies, the criminal justice system, and various governmental institutions at local, state, and federal level, such as the FDA, the Office of National Drug Control Policy (ONDCP), the Drug Enforcement Administration (DEA), and the Substance Abuse and Mental Health Administration (SAMHSA) (24).

The National Institute on Drug Abuse (NIDA) focuses its research toward new analgesics, alternative delivery systems and formulations, treatment of opioid abuse and addiction, and prevention of overdose deaths. The recommendations formulated stipulate the need for collaboration for prevention, education, and outreach (29, 33). The standard measure for pain assessment currently available may lack adequacy or accuracy. New evidence-based tools and technology platforms could help to deliver information on how to better treat various subpopulations of patients (34). Furthermore, registries should be set up to gather and process data related to various drugs, patient monitoring, expanded electronic medical records (EMR), and therapy. The theoretical framework is present. However, the concrete implementation of the collaboration between various healthcare actors involved in pain management and treatment of addiction in terms of (precompetitive) research, treatment, prevention, and education is not adequate to tackle the growing crisis (29).

More efforts are needed to investigate the development of curricular core competencies in the field of pain management and how to implement new educational approaches. Guidelines and best practices related to risk assessment and management as well as prescription behavior should be developed and implemented in new educational approaches (29). Collaborations should encompass education and training of multiple healthcare disciplines. This new educational models should be taught to the next generation of healthcare providers, including schools of medicine, nursing, physical therapy, and dentistry as well as part of a continuous learning track for all healthcare actors (29).

The search for innovative drugs with reduced abuse, tolerance, and addiction risk is situated in a more competitive environment and will require specific incentives for the pharmaceutical industry to collaborate (30, 32). Abuse-deterrent formulations are used in less than three percent of the cases, most likely due to their high price. Pharmaceutical companies might be reluctant to invest in developing them, when payers are not incentivized to cover such medication. PPPs, driven by the industry as they carry the burden for this crisis, should develop assisted funding systems for developing alternative drugs.

Investigating how to improve and speed up the regulatory system as well as pricing and reimbursement policies, is hence another key approach to collaborate with each other.

## Conclusion

The opioid crisis is of complicated and multifaceted nature that calls for urgent collaborative action. An interdisciplinary approach is needed by engaging several disciplines. Various types of PPPs should be set up to focus on research, training, and education. The research PPPs encompass early-phase research developing tools, technologies, and platforms to accelerate the drug development process, as well as the more competitive task of developing new pain medication and also defining the strategies, medication, and behavioral practices to treat opioid use disorders. Training and education partnerships aim to educate the various healthcare actors involved to implement these strategies and best practices in the healthcare system (32). The role of participation of patients, both those addicted due to physical and psychological pain, therein is pivotal.

PPPs are a tool, not an objective *per se*. The key stakeholders involved, each with their accompanying expertise, resources, and experiences are essential to implement the changes needed to tackle this public health crisis. Not one actor is solely responsible for all aspects of this opioid crisis, however, the classical roles of the various actors, such as the pharmaceutical industry, the nonprofit sector, academia, and the government needs to be reviewed with respect to the challenges faced (24). Successful collaborations are built upon trust, clear rules and agreements on the mission, the project objectives, the responsibilities of the different stakeholders, and a good definition of the key performance indicators that will be used to measure the PPP's success (24, 35). All need to work cooperatively to protect the public interest.

Regardless of promising outcomes of PPPs, the National Institute of Health (NIH) must remain critical and take ethical considerations into account when accepting any sort of support from companies of the e.g., pharmaceutical sector, since they have contributed to a large extent to the opioid crisis (14, 36).

It can be concluded that despite novel approaches and alternate formulations being developed to address the opioid addiction, more resources are needed and the urgent situation of the opioid crisis in the USA calls for more attention (32).

## Author Contributions

All authors listed have made a substantial, direct and intellectual contribution to the work, and approved it for publication.

### Conflict of Interest Statement

The authors declare that the research was conducted in the absence of any commercial or financial relationships that could be construed as a potential conflict of interest.

## Figures and Tables

**Graph 1:**
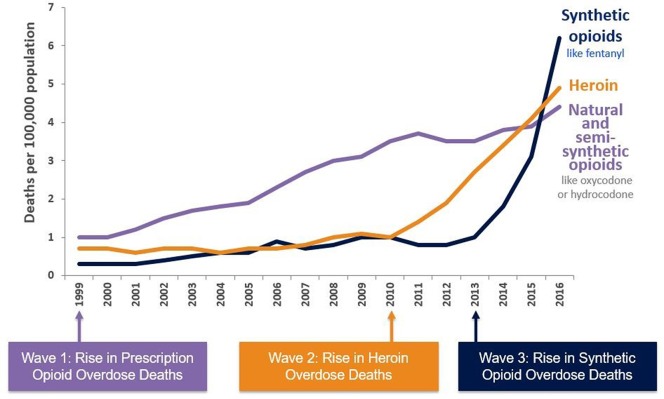
Three waves of the increasing opioid crisis (6).
